# An Observational Study Investigating Potential Risk Factors and Economic Impact for Bovine Ischaemic Teat Necrosis on Dairy Farms in Great Britain

**DOI:** 10.3389/fvets.2022.748259

**Published:** 2022-03-22

**Authors:** Hayley E. Crosby-Durrani, Roger W. Blowey, Al Manning, João Sucena Afonso, Stuart D. Carter, Nicholas J. Evans, Joseph W. Angell

**Affiliations:** ^1^Institute of Infection, Veterinary, and Ecological Sciences, University of Liverpool, Neston, United Kingdom; ^2^Retired, Gloucester, United Kingdom; ^3^Quality Milk Management Services Ltd., Wells, United Kingdom; ^4^Wern Vets CYF, Department of Research and Innovation, Ruthin, United Kingdom

**Keywords:** bovine, ischaemic, necrosis, questionnaire, risk factors, dairy

## Abstract

Bovine ischaemic teat necrosis (ITN) is an emerging disease of unknown aetiology that affects the teats of dairy cattle. It causes economic and animal welfare issues with many animals being culled. No effective treatments or epidemiological data to inform control strategies are currently available. The aim of this observational study was to investigate farmer-reported experiences and identify potential farm-level risk factors. In January 2018, a questionnaire was sent to a random sample of 1,855 Great Britain (GB) dairy farmers. A usable response rate of 12.3% was obtained. Fifty-one per cent [95% confidence interval (CI): 44.4–57.8%] of farmers reported having experienced ITN on their farm between 1985 and 2018. Rising numbers of farms indicated that ITN is an emerging disease with 46.3% of farmers reporting the first case in the 3 years up to 2018. At the animal level, 47.3% (95% CI: 38.7–55.9%) of the cases occurred during the first lactation and 78.9% (95% CI: 75.2–82.6%) within the first 90 days in milk. Only 20.8% (95% CI: 15.9–26.4%) of the cases were reported to recover, whereas 22.8% (95% CI: 17.8–28.5%) of the cases required culling. The remaining cases experienced complications such as loss of a teat and/or mastitis. From these data, the cost of ITN, through production losses and expenditure, was estimated to be £1,121 per farm per year. The costs were estimated at £720, £860 and £2,133 for recovered, complicated and culled cases, respectively. Univariable and multivariable logistic regression models were used to explore the associations between the presence of ITN on farm and various risk factors. The presence of udder cleft dermatitis (UCD) (odds ratio 2.80; 95% CI: 1.54–5.07; *p* < 0.01) and chapped teats (odds ratio 6.07; 95% CI: 1.96–18.76; *p* < 0.01) in the milking herd was associated with the presence of ITN at the farm level. This is the first national questionnaire of ITN within GB and highlights the association of UCD and chapped teats with ITN at the farm level. While there are many limitations and potential bias around farmer questionnaires, these findings highlight several key areas for further disease investigation and possible intervention.

## Introduction

Bovine ischaemic teat necrosis (ITN) is a relatively new disease, first reported in 2004 (1). The disease affects the teats of dairy cattle (*Bos taurus*) and can lead to sloughing of teat tissues, resulting in pain and discomfort, and consequently is a welfare problem (1). Also, ITN has economic consequences for farmers that have experienced this disease as many animals do not respond to treatment and have to be culled prematurely.

Ischaemic teat necrosis has been associated with the digital dermatitis (DD) *Treponema* bacteria (2) and thus is considered to potentially be infectious in nature. There are many infectious diseases that can affect the teat of the dairy cow. One of the differential diagnoses for ITN is bovine herpes mammillitis (BHM). Ischaemic teat necrosis and BHM can be differentiated based on their clinical presentations as ITN presents as a focal dry red to black area of necrosis on one or more teats (3) compared with the exudative lesion produced by BHM that can affect one teat or involve the entire udder (4, 5). Another different clinical presentation between the diseases is that ITN cases can be highly pruritic in nature (6), which is not a reported sign of BHM.

Some diseases of bovine udder skin are considered multifactorial and the result of the interactions of environmental, infectious and other factors. An example of such a disease is udder cleft dermatitis (UCD), lesions of which also reportedly contain DD *Treponema* spp. (7, 8). UCD typically affects the skin either in between the two halves of the udder or at the junction of the anterior udder and the abdomen (9–12). Clear aetiological, environmental and epidemiological data are lacking for ITN. Moreover, it is unknown how many GB dairy farms have experienced ITN and the associated cost implications of cases, although there are reports that ITN is an increasing problem (1, 2, 13). Hence, it is timely to identify how widespread this disease has become, its transmission dynamics, associated risk factors and the economic impact of ITN on the GB dairy industry.

Farmer questionnaires have been used many times to investigate potential areas of interest and risk factors associated with farm animal diseases (14–16). They have been used regularly in the dairy industry to gain further understanding of current farm practises and to identify how issues change over time (17–19). The aims of this study were to (1) investigate the farmer-reported experience of ITN on GB dairy farms, (2) identify potential risk factors and (3) calculate the management costs for a case of ITN by using a farmer-based postal questionnaire, with an online and telephone option.

## Materials and Methods

### Study Design

An observational study using a twelve-page postal questionnaire, with an additional pictorial guide of diseases affecting the bovine udder, was designed (see Supplementary Material).

### Sample Size Calculation

The study population was selected from producers designated as dairy farmers in a database of the Agricultural and Horticultural Development Board (AHDB). This board collects a levy from dairy farms in Great Britain (GB). The sample size was calculated using the online tool OpenEPi (https://www.openepi.com), and farms were randomly selected using simple randomisation to gain information across all types of dairy farms. There were 10,250 dairy farms in the database provided by AHDB Dairy in 2017, and for farmers to be eligible to complete the questionnaire, they had to be within this database and have an active milking cow dairy herd on a farm in GB. As the hypothesised frequency of ITN within the population of dairy farms was unknown, a value of 50% was used with confidence limits set at 5%. The sample size required to detect this value at a 95% confidence level for the GB dairy population was 371 dairy farms.

From publications that targeted the GB farming community, AHDB Dairy and author experience with questionnaire studies, a potential response rate was estimated to be 20% (14, 20). Therefore, to obtain a sample size of 371, 18.1% of the target population (1,855 questionnaires) was surveyed.

### Questionnaire Design

The aims of the questionnaire were to

1. Identify the proportion of farmers that have observed ITN on their farm and over what timeframe;
2. Gain information on when farmers reported the index ITN case on their farm;
3. Identify the reported at-risk animals (animal-level);
4. Investigate factors potentially associated with ITN at the farm level.

Farmers were asked to refer to the pictorial guide when answering disease-specific questions. The pictorial guide presented examples of different diseases described in the questionnaire for comparative purposes. This guide also included full written descriptions and was reviewed by farmers and industry experts (RB and AM) prior to the distribution of the postal questionnaire. This confirmed an accurate description of ITN and that farmers were readily able to correctly identify the other diseases affecting the teat skin from this guide. The images and written descriptions were also compared to veterinary textbooks (3, 6, 21). The questionnaire covered a wide range of topics including the following: questions related to the farmers' experience with ITN; the health of the udder; general animal health; milking routine; and the farm environment. Each question included a “don't know” and an “other” option. The “other” option had an area for free text to allow farmers to expand on their answers. As part of the questionnaire development, 26 dairy farmers were interviewed extensively during phone calls and farm visits to develop a pilot questionnaire. This pilot postal questionnaire was then distributed to 10 different dairy farmers. Five of the 10 farmers responded, and their feedback informed the final questionnaire design.

One week prior to questionnaire dispatch, a postcard stating that the farm will receive a postal questionnaire was sent. The questionnaire along with a cover letter and return envelope was posted in January 2018. Postal questionnaires included a link to an online version of the questionnaire and a telephone number in case farmers preferred to respond in that way or had questions that required clarification. All participants were given the option to withdraw from the study at any time and to self-select into a prize draw in appreciation of their time completing the questionnaire. The dataset was anonymised.

### Definition for a Case of ITN

An ITN-positive animal was an animal that had at least one teat lesion compatible with the working definition of ITN: a focal, dry, dark red to black well-demarcated area of necrosis on one or more teats, typically on the medial aspect of the teat extending to the udder. The lesion may or may not be pruritic. An ITN-positive farm was a farm with at least one animal recorded as presented with the lesion consistent with ITN.

### Data Analysis

A database was constructed with all questionnaire responses manually entered. After this, a series of range and consistency cheques were performed to identify any input errors and the retained hard copy of the questionnaire then consulted and any errors rectified. Many variables were categorical (Supplementary Table 1). Variables that were continuous in nature were transformed into categorical groups where appropriate. All analyses were carried out using R version 3.5.0 (22) using the following packages in alphabetical order: Amelia, base, DescTools, dplyr, lmtest, LogisticDx, Mass, PropCIs, ResourceSelection, sjPlot and stats.

Exploratory and descriptive statistical investigations were applied and the chi-squared test used to assess differences between groups. Logistic regression analyses were carried out where appropriate. For all analyses, statistical significance was set at *p* ≤ 0.05 for evidence of a strong association and p-value 0.05–0.2 for evidence of a weak association. The denominator changed per variable to reflect the number of farmers that responded to each question. Each farmer that responded to the questionnaire only represented a single farm, and so the term farmer or farm was used interchangeably.

Many variables contained some missing data, either where the participant had not answered, was unable to answer or where they had answered “don't know”. The pattern of missingness was assessed as a generalised pattern of missingness (23). As multiple imputation failed, where applicable, multivariable analyses were carried out on constrained datasets whereby observations with missing values were excluded from the model.

The primary outcome variable was the presence of ITN on the farm; secondary outcome variables were the presence of UCD and chapped teats.

### Cost of ITN

The costs associated with ITN were calculated using the questionnaire data alongside various industry guides and references. Costs were averaged over all calving systems and data used to calculate the cost per case. Three separate financial calculations were made based on the following categories: if the animal was an uncomplicated ITN case which recovered; if the cow lost the affected teat or developed mastitis; and, finally, if that animal was culled early on in the lactation due to ITN complications. For calculation purposes, it was assumed that once an ITN lesion appeared on the teat, milking the affected quarter would be challenging or not possible for the rest of the lactation. The reproductive losses were not calculated for a recovered case or a cull case of ITN but are included for a case with complications. It is assumed that a cull case was culled early in lactation, <100 days, due to the severity of the ITN lesion. For calculation purposes, a case was considered to affect only one teat and milk from the same quarter. Therefore, these are likely minimum costs as many reported cases affect more than one teat.

### Associations With ITN Presence on the Farm

Both univariable and multivariable analyses were carried out using logistic regression. Observations were excluded where farmers had not answered a question or had responded with “don't know”. All exposure variables with a *p* < 0.2 on univariable analysis were included for subsequent investigation within the multivariable regression models.

An initial multivariable model including all the selected exposure variables did not converge; consequently, variables were grouped into the following common themes: (1) disease factors: presence or absence of certain diseases on the farm; (2) chemical factors: such as disinfectant usage; and (3) farm environment and management factors: including other animals on the farm, vaccination history and calving system.

For each of the three themes, multivariable models were fitted using a stepwise backwards elimination strategy whereby a full model was fitted with all the selected variables for that category. Then, each variable was removed in turn and a likelihood ratio test carried out. Variables were retained if the resultant *p* < 0.05. Omitted variables were then added back in turn to the final model starting from the lowest p-value. A likelihood ratio test was performed after each addition and the variable retained in the model if *p* < 0.05. This process was continued until no further variables could be added to produce the final model.

Variables retained in each of these models were then combined in an overall model. Stepwise backwards elimination was carried out again as previously described using the explanatory variables from the previous three models to produce the final model.

The final model fit was assessed using the Hosmer–Lemeshow goodness-of-fit test and estimating the area under the receiver operating characteristic (ROC) curve. The mean predicted probability of the outcome (the presence of ITN on a farm) was then compared to the observed proportion of farms with that outcome to visually assess the reliability of the model.

The final multivariable model included two disease factors which potentially induced a risk of collider bias. To confirm this, the multivariable model was fitted without disease factors and variable with large numbers of missing observational values. However, such a multivariable model produced unreliable estimates and unrealistic standard errors; hence, univariable models are presented.

### Associations With UCD and Chapped Teats as Secondary Outcome Variables

From the results using ITN as the primary outcome variable, it was clear that UCD and chapped teats were associated with the presence of ITN on the farm. Given that the nature of the questionnaire data gathered was largely transferrable, the analysis was repeated using UCD and chapped teats as secondary outcomes. For UCD, a forward stepwise process was implemented as models did not converge when using a series of backwards approaches. As for ITN, there was the risk of collider bias; hence, multivariable models were fitted excluding all disease factors and variables with large amounts of missing data. Once again, multivariable models excluding disease factors produced unreliable estimates and unrealistic standard errors when using chapped teats as the outcome.

## Results

### Response Rate

Of the 1,855 questionnaires posted, 263 were returned including 256 in paper format, four online and three via email or telephone. All questionnaires were returned between January and March 2018. Of these, 228 were adequately completed, producing an overall returned response rate of 12.3% (95% CI: 10.8–13.9%). Response rates from each Devolved Nation (country) were similar with 12.3% of 225 (95% CI: 10.6–14.2%) respondents from England, 13.0% (95% CI: 8.5–18.7%) from Scotland and 13.3% (95% CI: 9.7–17.5%) from Wales. Three respondents did not indicate the country their farm was situated in. When using a 95% CI, there was no statistical difference in response rate per country with farmers from all countries reported having had cases of ITN. As not all answers in the questionnaire were completed, or farmers responded with the “don't know” response, the response rate per question varied. There were some redundancies within the sampling frame, and Table 1 shows the reported reasons for not completing the questionnaire.

**Table 1:** Reported response reasons for not completing the questionnaire.

**Reason**	**Number**
No longer in dairy farming	18
Not a dairy farm	4
No reason	2
Not the right address	1
Total	25

### Descriptive Statistics

**Figure 1:**
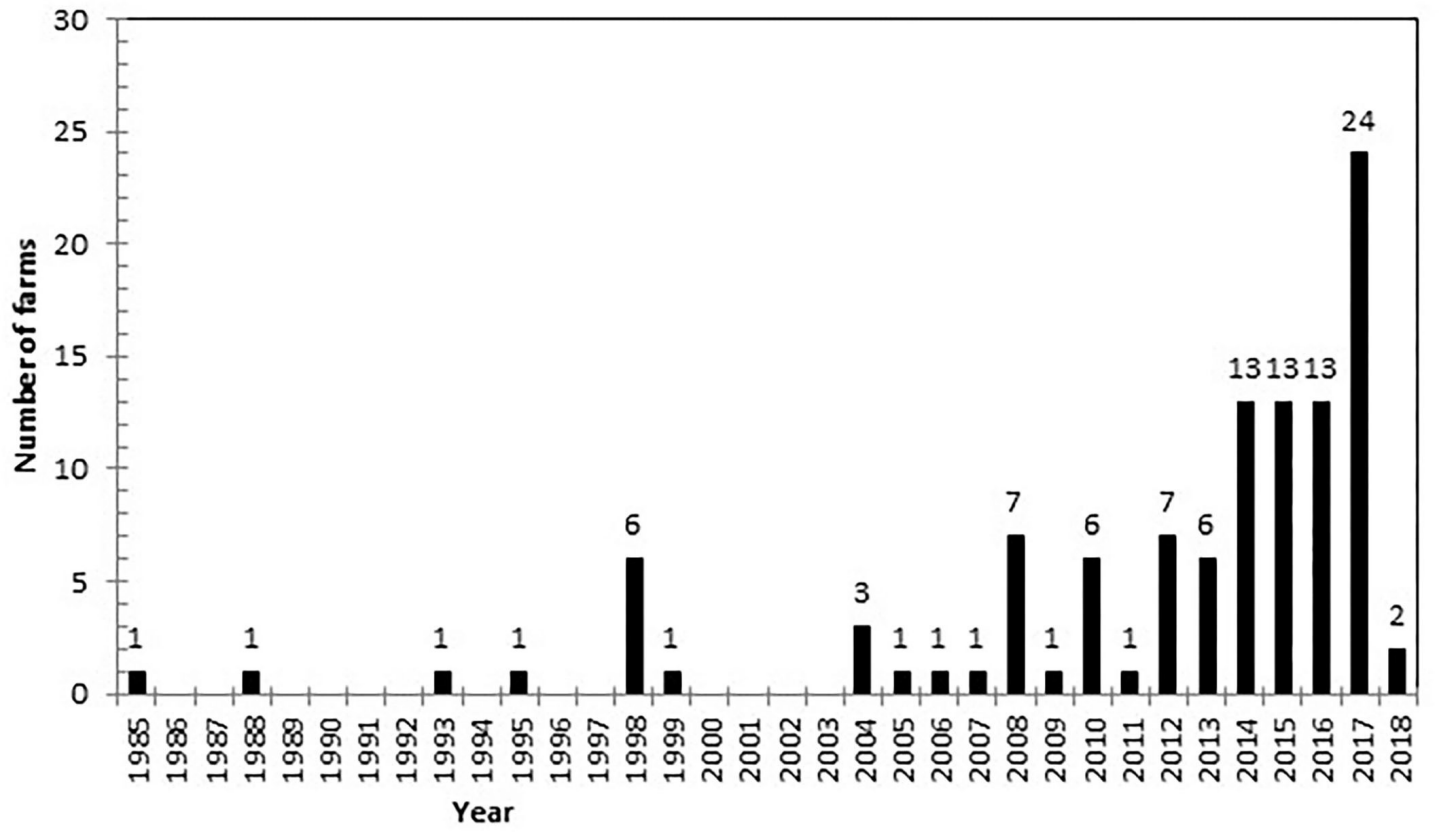
Frequency of the year farmers reported seeing the first case of ischaemic teat necrosis (ITN) on their farm. The number of farmers reporting the first case of ITN observed on the farm is persistently higher from 2012 than earlier years. Note there are only two farms reporting the first case in 2018 as the questionnaire was submitted in January 2018.

Out of 227 farmers, 116 (51.1%; 95% CI: 44.4–57.8%) reported that they had observed a case of ITN at some point between 1985 and 2018. Of those that provided a date when they first observed the disease on their farm (*n* = 108), fifty farmers (46.3%; 95% CI: 36.7–56.2%) reported seeing the first case of ITN in the 3 years up to 2018 (Figure 1). There was an increase in the number of farmers witnessing cases for the first time within the last decade.

Farms varied in size from 5 to 1,923 milking cows and were grouped into five categories: small, 5–100 milking cows (*n* = 45; 20.2%; 95% CI: 9.8–30.8%); small to medium, 101–140 milking cows (*n* = 45; 20.2%; 95% CI: 9.8–30.8%); medium, 141–200 milking cows (*n* = 51; 22.9%; 95% CI: 12.8–33.1%); medium to large, 201–300 milking cows (*n* = 52; 23.3%; 95% CI: 13.2–33.4%); and large, more than 300 milking cows (*n* = 30; 13.5%; 95% CI: 2.1–24.9%). These categories were devised so there were approximately similar numbers of farms in each category. All variable coding is provided in Supplementary Table 1. Of the 223 farmers that responded to the specific question, 171 (76.7%; 95% CI: 70.6–82.1%) farms had year round calving, 47 (21.1%; 95% CI: 15.9–27.0%) had seasonal calving systems and five (2.2%; 95% CI: 0.7–5.2%) had a combination of year round or seasonal patterns. When asked about housing, 28 of 226 respondents (12.4%; 95% CI: 8.4–17.4%) had lactating cows that were housed all year, 23 (10.2%; 95% CI: 6.6–14.9%) had cows at pasture all year and 175 (77.4%; 95% CI: 71.4–82.7%) had cows with pasture access and housing.

Participants also reported that they had previously called ITN by other names including teat sores, udder sores, cracked teats, dermatitis, “dermo”, sores, wart teats, black teat, teat scabs, manure burn, teat rot, cow pox, teat necrosis, orf, herpes mammillitis, “digi of the udder” and licking teat.

**Figure 2:**
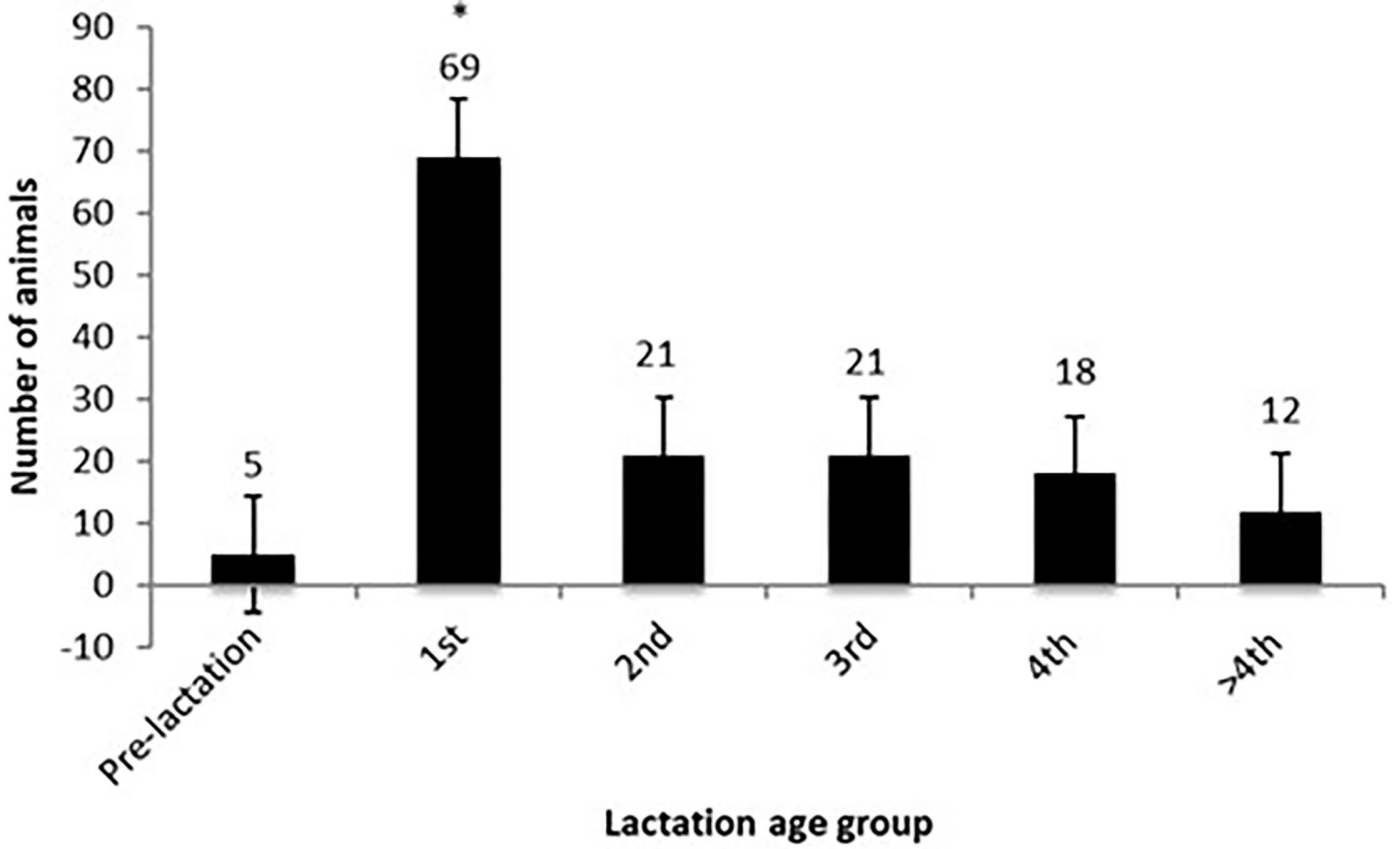
The production age of animals depending on the lactation the cow presented with an ischaemic teat necrosis (ITN) lesion on the teat. First lactation heifers are significantly over-reported as developing ITN lesions on their teats. *Represents a significant difference (*p* < 0.001).

To the question asking in which lactations the farmers had seen cases of ITN, 116 farmers responded, with 25 seeing ITN in more than one age group, therefore giving a total of 146 cases (Figure 2). The reported production age of animals indicated that first lactation cows were significantly more likely to develop ITN lesions with 47.3% (95% CI: 38.7–55.9%) of the cases in first lactation cows (*p* < 0.001) and <15% (95% CI: 0.8–29.2%) in any other lactation and only 3% (95% CI: −11.7–17.7%) pre-lactation.

**Figure 3:**
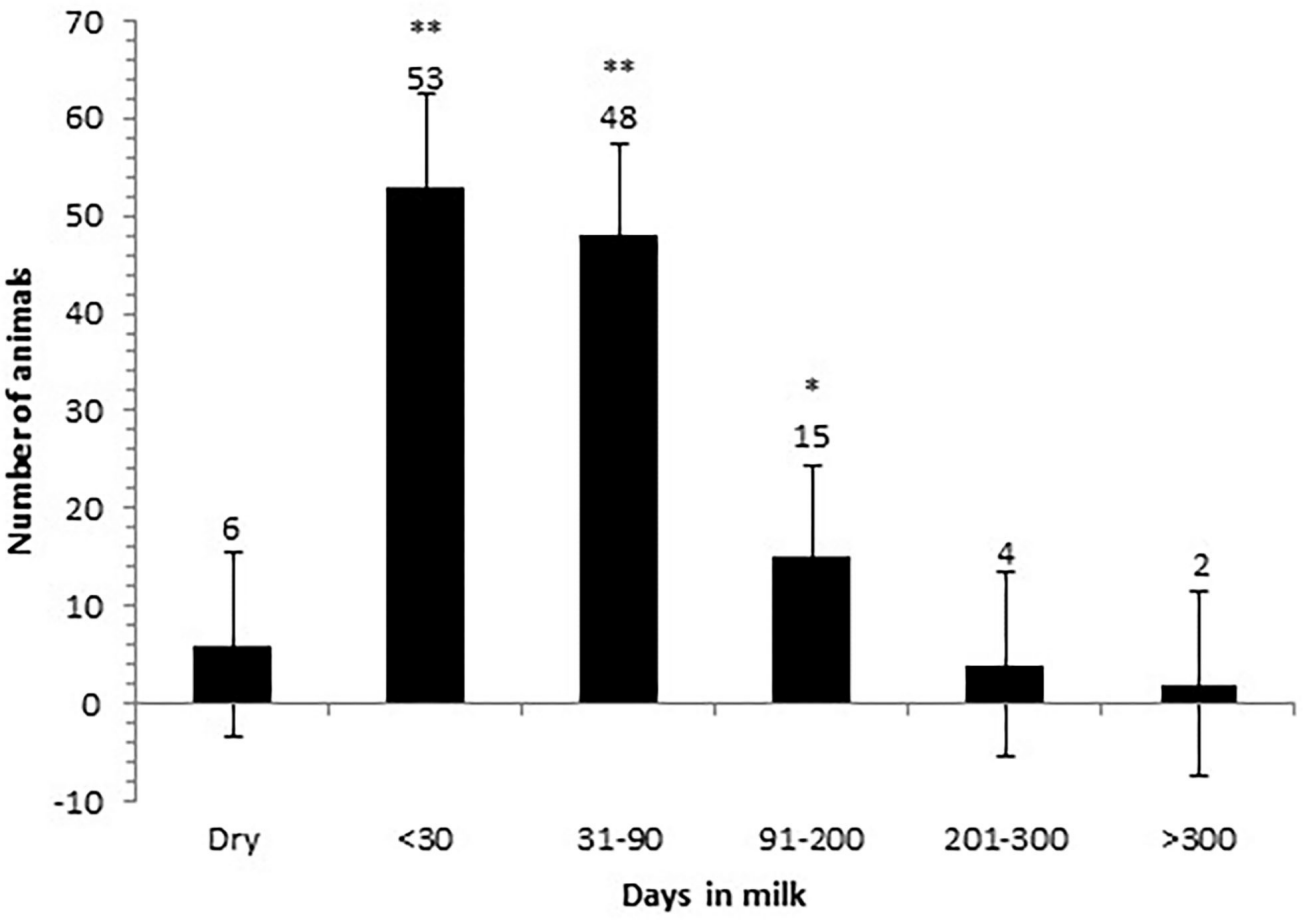
Days in milk that the affected cows are first observed with an ischaemic teat necrosis (ITN) lesion. The time period that cows are reported to first be observed with an ITN lesion on their teats are the categories of <30 days and 31–90 days in milk. Later in the lactation and during the dry period, cows are reportedly less likely to present with an ITN lesion. **Very strong evidence of a difference (*p* < 0.001); *strong evidence of a difference (*p* < 0.02).

Farmers also reported that there were significantly more animals affected by ITN lesions within the first 90 days in milk (DIM) (78.9%; 95% CI: 75.2–82.6%) compared to animals over 201 DIM and animals in the dry period (9.4%; 95% CI −6.4–25.2%; *p* < 0.001) (Figure 3). Seventeen farmers (14.8%; 95% CI: −0.9–30.5%) of 115 who responded reported the lesions appearing in more than one DIM category.

When questioned on the time of year that farmers observed ITN lesions, 116 farmers answered with 46 (39.7%; 95% CI: 28.7–50.7%) seeing the disease in more than one season for 225 cases of ITN. Farmers reported fewer cases during springtime compared with other seasons. There were 26 ITN cases (11.6%; 95% CI: 0–23.2%) reported in spring, 82 (36.4%; 95% CI: 28.1–44.7%) in summer, 66 (29.3%; 95% CI: 20.1–38.6%) in autumn and 51 (22.7%; 95% CI: 12.6–32.8%) in winter. However, once cofounding factors such as lactation number and calving pattern were investigated, models produced unreliable estimates.

To investigate the representation and similarity between the sampled study population and the GB dairy population, comparisons were made between the distributions of various characteristics in this study population and published figures for the GB dairy industry. Variables considered included mean herd size, average milk yield, rates of clinical mastitis, somatic cell count and proportion of farmers using seasonal and year-round calving systems. The estimate from this dataset was found to be broadly similar to the published GB data (Supplementary Table 2).

### Univariable Associations With the Presence of ITN on the Farm (Primary Outcome Variable)

Variables significantly associated with the presence of ITN are shown in Tables 2A–C. Other factors investigated are included as supplementary data (Supplementary Table 3).

**Table 2A:** Univariable “disease” associations with ischaemic teat necrosis (ITN) as the outcome variable.

**Variable (coding)**	**ITN + farms**	**ITN – farms**	**Odds ratio (lci-uci)**	***p*-value**
**Teat licking present on farm** ***n** **=*** **224**				
No teat licking (0)	28 (12.5%)	100 (44.6%)	^*^	-
Teat licking (1)	88 (39.3%)	8 (3.57%)	39.29 (17.02–90.67)	<0.01
**Presence of Bovine papilloma virus/warts** ***n** **=*** **217**				
No cases of bovine warts (0)	49 (22.6%)	66 (30.4%)	^*^	-
Cases of bovine warts (1)	61 (28.1%)	41 (18.9%)	2.00 (1.17–3.44)	0.01
**Presence of udder cleft dermatitis** ***n** **=*** **217**				
No cases of UCD (0)	59 (27.2%)	81 (37.3%)	^*^	-
Cases of UCD (1)	51 (23.5%)	26 (12.0%)	2.69 (1.51–4.81)	<0.01
**Presence of chapped teats** ***n** **=*** **217**				
No cases of chapped teats (0)	90 (41.5%)	103 (47.5%)	^*^	-
Cases of chapped teats (1)	20 (9.2%)	4 (1.8%)	5.72 (1.89–17.37)	<0.01
**Presence of DD in the summer** ***n** **=*** **212**				
Farms never had DD in summer (0)	50 (23.6%)	64 (30.2%)	^*^	-
Farms with DD in summer (1)	59 (27.8%)	39 (18.4%)	1.94 (1.12–3.35)	0.02
**Presence of DD in the autumn** ***n** **=*** **212**				
Farms never had DD in autumn (0)	21 (9.9%)	34 (16.0%)	^*^	-
Farms with DD in autumn (1)	88 (41.5%)	69 (32.5%)	2.06 (1.10–3.87)	0.02
**Type of mastitis present on the farm** ***n** **=*** **152**				
No testing for mastitis (0)	22 (14.5%)	38 (25.0%)	^*^	-
Environmental mastitis (1)	26 (17.1%)	25 (16.4%)	1.66 (0.78–3.55)	0.19
Contagious mastitis (2)	4 (2.6%)	6 (3.9%)	2.59 (0.66–10.19)	0.17
Mixed environmental and contagious (3)	9 (5.9%)	11 (7.2%)	2.11 (0.76–5.89)	0.15
Test but don't specify (5)	1 (0.66%)	3 (2.0%)	5.18 (0.51–52.90)	0.17

*The table shows the number of farms reporting each variable along with the proportion of farms in each ITN status (positive if they have cases of ITN, negative if they do not report cases of ITN), the odd's ratio and the p-value of the association of the variable to the ITN status. The number of farmers responding to each question varied with the number of farmers that answered (n). The numbers within the parenthesis next to each variable indicates the code used within the statistical models. The number of farms with or without the variable in question was recorded alongside the ITN status (±) with the percentage indicated in parenthesis. Odds ratio is indicated along with the Wald method of calculating the lower confidence interval (lci) and the upper confidence interval (uci). Variables with p > 0.05 are included as Supplementary Table 3*.

* *Indicates the reference group used for each variable*.

**Table 2B:** Univariable “chemical” factors associations with ischaemic teat necrosis (ITN) as the outcome variable.

**Variable (coding)**	**ITN + farms**	**ITN – farms**	**Odds ratio (lci-uci)**	***p*-value**
**Use of an automated dipping and flushing (ADF) system** ***n** **=*** **213**				
Don't use ADF (0)	74 (34.7%)	82 (38.5%)	^*^	-
Do use ADF (1)	37 (17.4%)	20 (9.4%)	2.05 (1.09–3.84)	0.03
**Disinfection of clustered between cows** ***n** **=*** **208**				
Don't disinfect clusters (0)	25 (12.0%)	47 (22.6%)	^*^	-
Disinfect clusters between every cow (1)	38 (18.3%)	29 (13.9%)	2.46 (1.24–4.89)	0.01
Disinfect cluster if mastitis/high SCC (2)	41 (19.7%)	28 (13.5%)	2.75 (1.39–5.45)	<0.01

*The table shows the number of farms reporting each variable along with the proportion of farms in each ITN status (positive if they have cases of ITN, negative if they do not report cases of ITN), the odd's ratio and the p-value of the association of the variable to the ITN status. The number of farmers responding to each question varied with the number of farmers that answered (n). The numbers within the parenthesis next to each variable indicates the code used within the statistical models. The number of farms with or without the variable in question was recorded alongside the ITN status (±) with the percentage indicated in parenthesis. Odds ratio is indicated along with the Wald method of calculating the lower confidence interval (lci) and the upper confidence interval (uci). Variables with p > 0.05 are included as Supplementary Table 3*.

* *Indicates the reference group used for each variable*.

**Table 2C:** Univariable management and milking machine factors associations with ischemic teat necrosis (ITN) as the outcome variable.

**Variable (coding)**	**ITN + farms**	**ITN – farms**	**Odds ratio (lci-uci)**	***p*-value**
**Presence of teat ringing after milking** ***n** **=*** **169**				
No teat ringing (0)	53 (31.4%)	65 (38.5%)	^*^	-
Cases of teat ringing (1)	32 (18.9%)	19 (11.2%)	2.07 (1.05–4.05)	0.03
**Presence of teat end keratosis** ***n** **=*** **169**				
No teat end keratosis (0)	36 (21.3%)	56 (33.1%)	^*^	-
Cases of teat end keratosis (1)	49 (29.0%)	28 (16.6%)	2.72 (1.46–5.09)	<0.01
**Foremilk cows before milking** ***n** **=*** **224**				
Don't foremilk (0)	9 (4.0%)	22 (9.8%)	^*^	-
Yes, always foremilk (1)	42 (18.8%)	29 (12.9%)	3.54 (1.42–8.78)	0.01
Foremilk most of the time (2)	12 (5.4%)	14 (6.3%)	2.10 (0.70–6.25)	0.19
Foremilk occasionally (3)	14 (6.3%)	17 (7.6%)	2.01 (0.70–5.75)	0.19
Foremilk if suspect mastitis (4)	38 (17.0%)	27 (12.1%)	3.44 (1.37–8.63)	0.01
**Site of heifer rearing for the farm** ***n** **=*** **220.7**				
Heifers are reared on the same site (1)	82 (37.3%)	62 (28.2%)	^*^	-
Heifers reared on the same farm but different site (2)	21 (9.5%)	31 (14.1%)	0.51 (0.27–0.98)	0.04
Reared on different farm (3)	7 (3.2%)	10 (4.5%)	0.53 (0.19–1.47)	0.22
**Freshly calved cow management** ***n** **=*** **216**				
Fresh cows housed year round (1)	25 (11.6%)	12 (5.6%)	^*^	-
Fresh cows housed at night (2)	17 (7.9%)	12 (5.6%)	0.68 (0.25–1.87)	0.45
Fresh cows housed in winter (3)	52 (24.1%)	59 (27.3%)	0.42 (0.19–0.93)	0.03
Fresh cows housed at night and in winter (4)	10 (4.6%)	9 (4.2%)	0.53 (0.17–1.66)	0.28
Fresh cows at pasture year round (5)	9 (4.2%)	11 (5.1%)	0.39 (0.13–1.20)	0.10
**Freshly calved cow housing** ***n** **=*** **216**				
Fresh cows in cubicle housing (1)	44 (20.4%)	58 (26.9%)	^*^	-
Fresh cows in loose housing (2)	50 (23.1%)	30 (13.9%)	2.20 (1.21–4.00)	0.01
Fresh cows cubicles and loose housing (3)	16 (7.4%)	13 (6.0%)	1.62 (0.71–3.72)	0.25
Fresh cows no housing (4)	2 (0.93%)	3 (1.4%)	0.88 (0.14–5.49)	0.89
**Freshly calved cows bedded on straw** ***n** **=*** **210**				
Fresh cows not on straw (0)	34 (16.2%)	47 (22.4%)	^*^	-
Fresh cows on straw (1)	75 (35.7%)	54 (25.7%)	1.92 (1.09–3.37)	0.02
**Heifer housing** ***n** **=*** **207.2**				
Heifers in cubicles (1)	49 (23.7%)	35 (16.9%)	^*^	-
Heifers in loose housing (2)	25 (12.1%)	39 (18.8%)	0.46 (0.24–0.89)	0.02
Heifers in cubicles and loose (3)	27 (13.0%)	20 (9.7%)	0.96 (0.47–1.99)	0.92
No housing (4)	7 (3.4%)	3 (1.4%)	1.67 (0.40–6.90)	0.48
**Time calves with dams** ***n** **=*** **221**				
0-1 hours (1)	3 (1.4%)	11 (5.0%)	^*^	-
1 <12 hours (2)	47 (21.3%)	29 (13.1%)	5.94 (1.53–23.10)	0.01
12 <24 hours (3)	27 (12.2%)	26 (11.8%)	3.81 (0.95–15.22)	0.06
24 <48 hours (4)	20 (9.0%)	18 (8.1%)	4.07 (0.98–16.97)	0.05
>48 hours (5)	19 (8.6%)	21 (9.5%)	3.32 (0.80–13.72)	0.10
**Average number of dry cows for year round calving systems** ***n** **=*** **219**				
1–20 dry cows (1)	47 (21.5%)	48 (21.9%)	^*^	-
21–40 dry cows (2)	32 (14.6%)	28 (12.8%)	1.17 (0.61–223)	0.64
41–65 dry cows (3)	10 (4.6%)	3 (1.4%)	3.40 (0.88–13.15)	0.08
65+ dry cows (4)	3 (1.4%)	1 (0.46%)	3.06 (0.31–30.52)	0.34
**Number of cows in milk for year round calving systems** ***n** **=*** **220**				
1–50 cows in milk (1)	4 (1.8%)	8 (3.6%)	^*^	-
51–100 cows in milk (2)	23 (10.5%)	25 (11.4%)	1.84 (0.49–6.94)	0.37
101–150 cows in milk (3)	17 (7.7%)	20 (9.1%)	1.7 (0.43-6.65)	0.45
151–200 cows in milk (4)	23 (10.5%)	12 (5.5%)	3.83 (0.96–15.37)	0.06
201-250 cows in milk (5)	8 (3.6%)	10 (4.5%)	1.6 (0.35–7.30)	0.54
251–300 cows in milk (6)	9 (4.1%)	4 (1.8%)	4.5 (0.84–24.18)	0.08
301+ cows in milk (7)	9 (4.1%)	1 (0.45%)	18 (1.65–196.28)	0.02
**Average milk yield/cow/year** ***n** **=*** **216**				
<6,000 litres	17 (7.9%)	22 (10.2%)	^*^	-
6,001–8,000 litres	28 (13.0%)	44 (20.4%)	0.82 (0.37–1.82)	0.63
8,001–10,000 litres	53 (24.5%)	30 (13.9%)	2.29 (1.05–4.96)	0.04
>10,001 litres	14 (6.5%)	8 (3.7%)	2.26 (0.77–6.63)	0.14
**Milking herd size** ***n** **=*** **223**				
Small milking herd (1)	15 (6.7%)	30 (13.5%)	^*^	-
Small to medium milking herd (2)	22 (9.9%)	23 (10.3%)	1.91 (0.82–4.49)	0.14
Medium milking herd (3)	29 (13.0%)	22 (9.9%)	2.64 (1.15–6.05)	0.02
Medium to large milking herd (4)	28 (12.6%)	24 (10.8%)	2.33 (1.02–5.33)	0.04
Large milking herd (5)	21 (9.4%)	9 (4.0%)	4.67 (1.72–12.65)	<0.01
**Total herd size** ***n** **=*** **223**				
Small total herd (1)	12 (5.4%)	29 (13.0%)	^*^	-
Small to medium total herd (2)	25 (11.2%)	21 (9.4%)	2.88 (1.18–6.99)	0.02
Medium total herd (3)	24 (10.8%)	20 (9.0%)	2.9 (1.18–7.11)	0.02
Medium to large total herd (4)	21 (9.4%)	18 (8.1%)	2.82 (1.12–7.08)	0.03
Large total herd (5)	33 (14.8%)	20 (9.0%)	3.99 (1.67–9.54)	<0.01

*The table shows the number of farms reporting each variable along with the proportion of farms in each ITN status (positive if they have cases of ITN, negative if they do not report cases of ITN), the odds ratio and the p-value of the association of the variable to the ITN status. The number of farmers responding to each question varied with the number of farmers that answered (n). The numbers within the parenthesis next to each variable indicates the code used within the statistical models. The number of farms with or without the variable in question was recorded alongside the ITN status (±) with the percentage indicated in parentheses. Odds ratio is indicated along with the Wald method of calculating the lower confidence interval (lci) and the upper confidence interval (uci). Variables with p > 0.05 are included in Supplementary Table 3*.

* *Indicates the reference group used for each variable*.

Of 117 possible variables, 23 were strongly associated with the presence of ITN on a farm (*p* < 0.05), and a further 30 variables were weakly associated (*p* < 0.2). These variables included other diseases (Table 2A), chemical factors (Table 2B), management and milking machine factors (Table 2C).

### Multivariable Analysis

**Figure 4:**
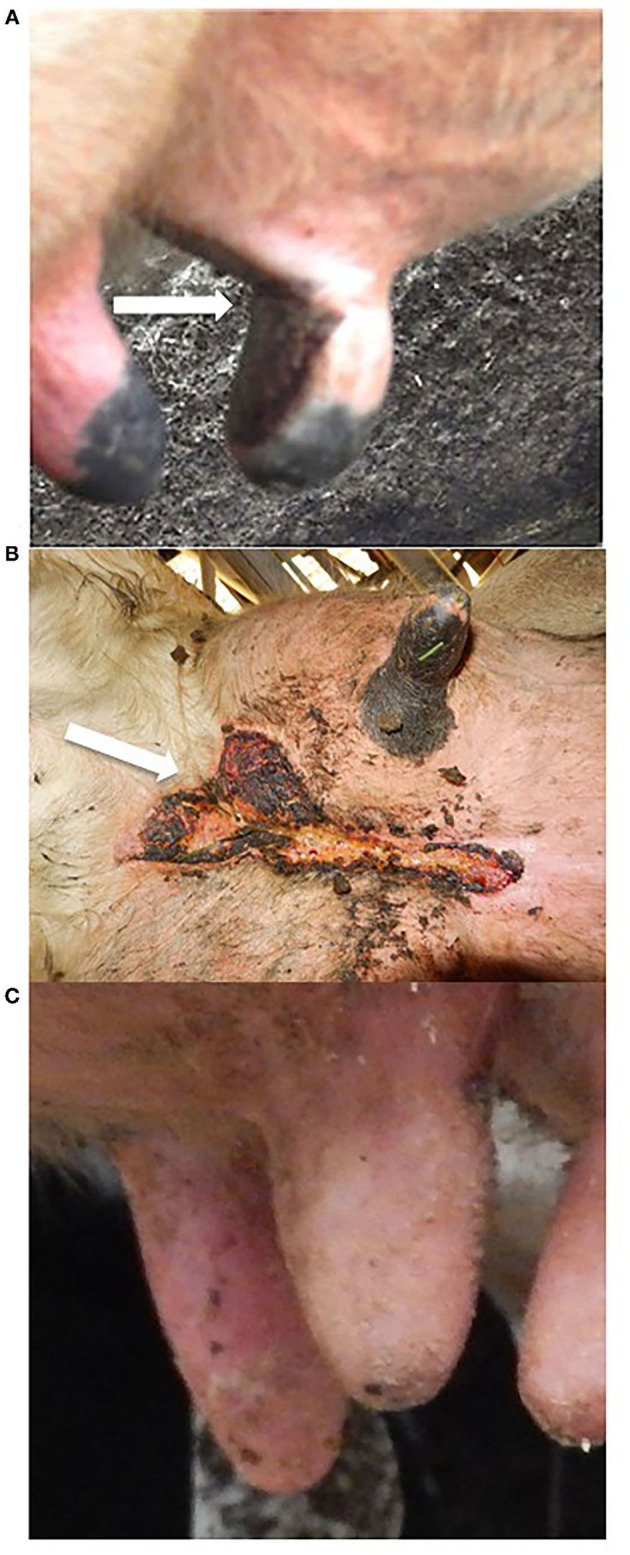
Udder lesions. **(A)** Photograph of a typical ischaemic teat necrosis (ITN) lesion with dark red to black, well-demarcated area of necrosis on the medial aspect of the teat extending to the udder indicated by the arrow. **(B)** Photograph of a typical udder cleft dermatitis (UCD) lesion affecting midline between the two halves of the udder and cranially to the cleft between the anterior udder and the abdomen indicated by the arrow. **(C)** Photograph of chapped teats with rough, dry skin on over the entire teats.

The final multivariable model included the presence of UCD (OR: 2.80; 95% CI: 1.54–5.07; *p* < 0.01) and chapped teats (OR: 6.07; 95% CI: 1.96–18.76; *p* < 0.01) on the farm (Table 3). Figure 4 demonstrates typical presentations of UCD and chapped teats.

**Table 3:** The final multivariable model with the reported presence of ischaemic teat necrosis (ITN) on the farm as the outcome variable.

**Variable**	**Odds ratio (lci-uci)**	***p*-value**
Intercept	0.61^*^	-
UCD	2.80 (1.54–5.07)	<0.01
Chapped teats	6.07 (1.96–18.76)	<0.01

*Indicates strong ITN associations with udder cleft dermatitis (UCD) and chapped teats (n = 217 farms). Wald's method was used to calculate the lower confidence interval (lci) and the upper confidence interval (uci) and is indicated in parentheses next to the value for the odds ratio. UCD, udder cleft dermatitis on the farm*.

* *Indicates the reference group used for each variable*.

For this model, the Hosmer–Lemeshow goodness-of-fit test was 0.96, and the area under the receiver operating characteristic (ROC) curve was 0.67 (0.60–0.73) and indicated evidence of a good fit. Where possible, visual comparisons of the mean predicted and observed percentages of farms with ITN were carried out. Each combination of the explanatory variables from the model was similar, and examination of the 95% CIs revealed no significant differences (Supplementary Table 4).

### The Cost of ITN

One hundred and eight farmers reported the clinical outcomes of 250 ITN cases. Fifty-two cases recovered (20.8%; 95% CI: 15.9–26.4%) and 57 were culled (22.8%; 95% CI: 17.8–28.5%). The remaining 141 cases (56.4%; 95% CI: 50.0–62.6%) either lost the teat and were milked on reduced numbers of teats or the cow subsequently developed mastitis. Costs associated with loss of production, treatment costs, visits by veterinary surgeons, extra labour costs and, where required, the cost of a replacement animal were calculated based on these three clinical outcomes. Performance averages were obtained from across all calving patterns in the dataset and compared with industry standards and literature in similar fields (Tables 4A–D).

**Table 4A:** The estimated cost of a case of ischaemic teat necrosis (ITN).

**Component**	**Breakdown**	**Cost**	**Source**
Milk yield/lactation	8,000/litre	-	Dataset (24)
Milk yield /quarter/ day	6.15 litres	-	Dataset
Price per litre of milk	£0.28		(24, 25)
Length of lactation	325 days	-	Dataset
ITN lesion onset	25 DIM	-	Dataset

*Breakdown of the components and assumptions used for the calculations. The source or reference used to devise these calculations is also indicated in the table. These key figures were used to calculate the costs in Tables 4B–D*.

*DIM, days in milk; £, pounds Sterling*.

**Table 4B:** The estimated cost for an uncomplicated case of ischaemic teat necrosis (ITN) that recovers.

**Component**	**Breakdown**	**Cost**	**Source**
Milk loss from ¼ for 300 days	£0.28 x 6.15 x 300	£516.60	Dataset (25)
Vet visit & medicines	£80 + £45	£125	
Milk loss for 7 day withdrawal period	£0.28 x 24.6 x 7	£48.22	Dataset (25)
Extra labour costs for a case of ITN^*^	£8.72/h x 0.5 x 7	£30.52	(24, 26)
**Total costs for an uncomplicated ITN case that recovered**			**£720.34**

*The calculations utilise the assumptions displayed in Table 4A. The source or reference used to devise these calculations is also indicated in the table*.

* *Extra labour costs calculated by assuming and a case requires an extra 30 min a day for 7 days. h, hour*.

**Table 4C:** The estimated cost for a complicated case of ischaemic teat necrosis (ITN) that lost the teat and/or developed mastitis.

**Component**	**Breakdown**	**Cost**	**Source**
Average costs for a case of mastitis	$453.17,$:£ 0.76	£344.41	(27–29)
Costs to be excluded^*^; Vet fees and medicines; Milk loss (withdrawal period); Extra labour costs	£125£48.22; £30.52	–£203.74	
**Total cost for a complicated case of ITN**	£720 + £342.45 – £203.74		**£860.67**

*The calculations utilise the assumptions displayed in Table 4A. The source or reference used to devise these calculations is also indicated in the table. One reference the currency was in US dollars, and thus the exchange rate used to calculate the cost in pounds sterling is shown*.

* *As included with the cost for a case of mastitis. $, US dollar; £, pounds sterling; $:£, US dollar to pounds sterling exchange rate*.

**Table 4D:** The estimated cost for a case of ischaemic teat necrosis (ITN) that required culling before the end of lactation.

**Component**	**Breakdown**	**Cost**	**Source**
Replacement animal^*^		£1,500	(30)
Average value back from the cull cow^**^		–£400	(24, 26, 30)
Extra loss of milk if culled before 100 DIM	200 DIM x0.28x¾ 24.6	£1,033.20	
**Total cost for a cull case**		£2133.20^***^	

*The calculations utilise the assumptions displayed in Table 4A. The source or reference used to devise these calculations is also indicated in the table*.

* *Replacement animal is the cost of a first lactation animal in a year-round calving pattern*.

** *Assuming the carcase is acceptable for slaughter and meat production*.

*** *Does not include any costs for treatments. DIM, days in milk; £, pounds sterling*.

For cows experiencing ITN, 20.8% recovered, 22.8% were culled and 56.4% had complications. Therefore, the cost per case varied, depending on the outcome, between £720.34 and £2,133.02. To calculate the average cost per farm per year, the probability of each clinical outcome was multiplied by the cost of the outcome and combined to give an average cost per case per farm per year of £1,121.62. This was a minimum figure as it was assumed that each farm would experience only a single case of ITN each year.

### Associations With the Presence of UCD on the Farm

Univariable analysis with UCD as the outcome variable revealed strong associations with 93 variables (*p* ≤ 0.05) and weak associations with further 12 variables (*p*-value: 0.05–0.2) (Supplementary Table 5). As with ITN, the associated variables were from all three categories (disease, chemical and farm management factors). The final multivariable model included three parameters, namely the presence of ITN on the farm, having lactating cows bedded on sawdust and cases of teat end eversion after milking, all of which were associated with an increased likelihood of reporting cases of UCD on the farm (Table 5).

**Table 5:** The reported associations with presence of udder cleft dermatitis (UCD) on the farm: final multivariable model with UCD as the outcome variable (*n* = 158).

**Variable**	**Odds ratio (lci-uci)**	**p-value**
Intercept	0.66^*^	-
ITN	3.14 (1.42–6.97)	0.01
Lactating cows bedded on sawdust	2.94 (1.37–6.29)	0.01
Teat end eversion	3.05 (1.06–8.77)	0.04
Calves with dams:		
1–12 h	0.12 (0.027–0.54)	0.01
12–24 h	0.41 (0.095–1.75)	0.23
24–48 h	0.33 (0.074–1.47)	0.15
>48 h	0.089 (0.017–0.46)	<0.01

*Wald's method was used for calculating the lower confidence interval (lci) and the upper confidence intervals (uci) and is indicated in parentheses next to the value for the odds ratio*.

*ITN, ischaemic teat necrosis on the farm; OR, odds ratio*.

* *Indicates the reference group used for each variable*.

For this model, the Hosmer–Lemeshow goodness-of-fit test was 0.80, and the area under the ROC curve was 0.76 (0.68–0.83) implying that the model was a good fit of the data. Due to the added number of variables in this model and the complexities of the variables, the predicted percentage probabilities are not presented for these data.

A multivariable model excluding disease variables and variables with large amounts of missing data was fitted (supplementary multivariable UCD model). This multivariable model included the following variables: type of housing used for lactating cows, if lactating cows were bedded on sawdust, the average milk yield per cow per year and if there was no isolation period on the farm when introducing new animals. The Hosmer–Lemeshow goodness-of-fit test was 0.69, and the area under the ROC curve was 0.78 (0.71–0.84) indicating that the model was a fair fit of the data.

### Association With Presence of Chapped Teats on the Farm

Univariable analysis with chapped teats as the outcome variable revealed strong associations with 97 variables and weak associations with two variables (Supplementary Table 6). The final multivariable model contained two variables (Table 6).

**Table 6:** The reported associations with chapped teats as the outcome variable (*n* = 101 farms).

**Variable**	**Odds ratio (lci-uci)**	***p*-value**
Intercept	0.04^*^	-
Peracetic acid in pre dip	8.91 (2.06–38.59)	<0.01
Use an ADF system	4.04 (1.04–15.69)	0.04

*Wald's method was used for calculating the lower confidence interval (lci) and upper confidence intervals (uci) and is indicated in parentheses next to the value for the odds ratio*.

*ADF, automated dipping and flushing system is used during milking*.

* *Indicates the reference group used for each variable*.

The Hosmer–Lemeshow goodness-of-fit test was 0.71, and the area under the ROC curve was 0.73 (0.58–0.90) indicating that the model was a fair fit of the data. The probability of reporting a case of chapped teats on the farm was predicted from the final model and compared to the observed probability of having chapped teats on the farm; these were very similar (Supplementary Table 7).

## Discussion

### Descriptive Statistics

Ischaemic teat necrosis is a disease which poses an important and increasing challenge for the dairy industry but has not been well studied (2). This is the first national study that investigated farmer experiences of ITN within GB. This study has revealed some key foundations and hypotheses for further investigation. In particular, ITN was reported on over half of GB dairy farms between 1985 and 2018. Furthermore, farms from all parts of GB reported cases, and there were no differences in reporting between geographical countries. This high proportion as well as reports from across GB is concerning particularly as this study identified that the number of farms experiencing the disease for the first time appears to have increased in recent years. Hence, based on these data, ITN could be considered already endemic in GB, although given the continued yearly increases reported in this study, it could also be designated as emerging.

To investigate the generalisability of these data to the rest of the GB dairy population, various analyses were carried out. In this study, just over three quarters of farmers stated that their farm had an all-year-around calving system, while about a fifth were seasonal and 2% had a combination of the 2 systems with one group of cows following a seasonal pattern and the remaining cows following year-round systems. This is similar to the reported demographic approximation whereby 85% of the GB dairy farmers report as having all-year-round calving systems (24). The apparent difference may be due to the increasing popularity to move to seasonal farms in GB to improve efficiency (31). Nevertheless, all-year-round calving systems predominate, and this gives further confidence that this study aligns with and is representative of the GB dairy population. Additional comparisons were made using other variables, demonstrating the similarities of the study dataset with available published data for the GB dairy population.

Considering the question of whether the farmers knew the ITN lesions by another name, it was clear that there were misunderstandings around the identification of the individual diseases that affect the bovine udder, and for this reason, the pictorial guide accompanying the questionnaire was essential to raise awareness of different lesions and their associated names, as well as to ensure accuracy when answering questions in relation to a specific lesion. From farmer interviews, the authors identified that farmers could readily distinguish between teat skin diseases using this guide. Farmers were encouraged and made contact to discuss questions if they were unsure how to answer. Inevitably, this is not an ideal format to obtain such information as it can introduce observational and misclassification bias. However, the use of pictorial guides to aid farmer questionnaires is a well-established methodology to ensure collection of reliable data (14, 32).

As with all questionnaires, there is the potential for reporting bias as farmers that have seen the disease may be more likely to respond, and there is also the issue of recall bias when asked to think of an event in the past (33). There is a suggestion of recall bias in the data where there are apparent peaks in cases in 1998 and 2008 (20 and 10 years before the questionnaire). The responses may also have been biassed depending on the length of time the farmer had been actively farming. If the dataset contained more farmers with a shorter history on a dairy farm, then a case presenting on the farm for the first time is more likely to be bias towards recent years. Unfortunately, the data on the length of time a farmer had been farming were not captured and therefore is a weakness in the study. The overall response rate in this study was lower than anticipated which was partly due to redundancies within the sampling frame. The questionnaire was lengthy, and this may have discouraged some potential participants. In addition, a follow-up reminder with a random selection of farmers called for a telephone interview to discuss their answers was planned to increase the response rate, but due to unforeseen circumstances, this did not occur. However, there were still a substantial proportion, almost half, of farmers who responded who had not seen the disease. It is also possible that responses were motivated by farmer desire to gain further knowledge, or from the understanding of the potential devastating effects ITN could have if it occurred on their farm.

The potential for collider bias was explored within this dataset. Collider bias happens when the outcome of the variables can affect the likelihood of being sampled (34). In this study, both ITN and UCD are skin diseases of the udder, and this may cause farmers who have experienced one or the other to self-select to complete the questionnaire. Unfortunately, this cannot be mitigated for entirely with voluntary farmer-based observational studies. However, to explore the possibility of the presence of collider bias, a comparison of key variables within the dataset was made with those of existing published studies. These analyses demonstrated that whilst this study represents a small sample of the GB dairy farmer population, the sample farms were broadly similar in terms of milking herd size, average milk yield, rates of clinical mastitis and average yearly somatic cell count. As such, whilst the possibility of collider bias cannot be totally eliminated, it is not readily apparent within this study at this stage. Additionally, multivariable models without disease factors were constructed to reduce the risk of collider bias in the analysis. However, it was not possible to fit multivariable models with reliable estimates and realistic standard errors for ITN or chapped teats as an outcome; therefore, the data presented in the univariable analyses are recommended for future investigations.

From the data presented, there are several important findings that may be worth pursuing as potential intervention strategies. For example, the finding at the animal level, first lactation animals in the first 90 days in milk appear to be the group most at risk of ITN development. It is vital that this is followed up with further longitudinal studies as this information could be utilised to encourage regular careful inspection of the teats in these animals at every milking to identify the disease early on in its clinical presentation. There are many studies that encourage the monitoring of early lactation animals for clinical mastitis (potentially affecting profitability), which indicate infections acquired in the dry period (17, 27, 28, 35). The same measures could aid in the rapid detection of ITN and its control.

### Economic Implications of ITN

In this study, farmers reported that slightly more than a fifth of cows with ITN were culled and only around a fifth recovered, and the remaining cases had complications such as teat loss and/or mastitis. This set of outcomes not only is important for animal welfare but also has an economic impact. A recovered case of ITN is estimated to cost £720, a complicated case to cost around £859 and a culled case to cost at least £2,992. Therefore, the average cost per farm, taking into consideration the expected proportions of each clinical presentation and assuming one case per farm per year, was estimated to be £1,121. This is similar to the study by Down et al. (36), whereby the costs associated with clinical mastitis were investigated; the costs of both diseases increase substantially when a cow is culled. Given that 22.8% of ITN cases require culling, many of them first lactation heifers, this is likely to be a substantial loss for farmers not only in monetary terms but also in genetic potential. Due to the reported increasing numbers of cases observed over the last few years and due to increasing costs of treatment, the number appears likely to increase with each year.

### Potential Farm-Level Risk Factors for ITN

Regression analysis of questionnaire data has been utilised frequently to identify potential farm-level risk factors for diseases (14, 15, 37). In this study, if the farm had cases of UCD or cases of chapped teats on the farm, then farmers were more likely to have reported a case of ITN. The predicted probabilities from the multivariable models demonstrated the likelihood of reporting ITN when either UCD or chapped teats are presented individually or in combination. Multiple methods were applied to denote confidence in these models showing that UCD and chapped teats were important factors associated with ITN that warrant further investigation. These associations may have a causal or reverse causal link, or may reflect some third factor not detected in this study.

To reduce the risk of collider bias within the models, and also bias due to missingness, multivariable models excluding disease factors and those variables with large amounts of missing data were constructed. However, they produced unreliable estimates and unrealistic standard errors; therefore, univariable model estimates are presented for scrutiny.

### Potential Farm-Level Risk Factors for UCD and Chapped Teats

The authors investigated potential farmer-reported farm-level risk factors for reporting cases of UCD and chapped teats. Although the original questionnaire was not designed for such investigation, due to the nature of the questions asked, it was deemed a logical approach to analyse the data to investigate these notable diseases and investigate potential farm-level risk factors for both and consequently identify additional potential areas for intervention. Udder cleft dermatitis and ITN were strongly related as both appeared as potential farm-level risk factors for each other. However, chapped teats were more associated with chemical factors, specifically the use of peracetic acid in a pre-milking formulation and the use of some form of automated dipping and/or flushing system. Compared to the model for ITN, the number of observations was reduced for these models as a result of missing values. As such, validation tables were used to assess if there was an important amount of missing data for the farms with and without the disease. There were no significant differences identified due to missing data, and the pattern of missingness was mostly a generalised pattern of missingness (23). Therefore, the models were unlikely to have been biassed in this manner.

The findings of ITN and UCD as potential farm-level risk factors for each other were biologically plausible and may indicate a common underlying aetiopathogenesis. It is also common amongst the medical and veterinary fields to find an infectious or non-infectious disease process which will predispose to another disease; for example, many bacterial pneumonias will be preceded by a viral respiratory infection (38–40). Whilst submission bias could skew associations, these reported risk factors warrant further investigation.

### ITN and Other Diseases

In this study, there was no association of ITN with DD. The reported hypothesis that ITN is associated with DD treponemal bacteria may not hold true, and further work is needed to clarify this area (2). From the model investigating UCD as the outcome variable, it was hypothesised that lactating cows that were bedded on sawdust and the presence of teat end eversion in lactating animals within the milking herd on the farm also increased the likelihood of developing UCD and thus potentially ITN. Studies in the Netherlands and Sweden have identified risk factors for UCD such as conformational traits at an individual level, the use of a foot bath, high-producing herds, breed and housing factors at a farm level (10, 11, 41). This study has highlighted potential differences in risk factors for UCD between GB and other countries.

As there was also the potential for collider bias with the model using UCD as the outcome variable, a multivariable model excluding disease factors and variables with large numbers of missing observation was fitted with similar reliability to the model including these excluded variables. The variables in this model included the type of housing that lactating cows are in, with farmers that have lactating cows without housing more likely to report cases of UCD. Cows bedded on sawdust and higher-yielding herds with no isolation periods are also more likely to report cases of UCD, which is consistent with the findings in the Netherlands and Sweden (10, 11, 41). These findings require further investigation as they may lead to farmers being able to reduce cases of UCD on their farms.

The final model investigating factors associated with the presence of chapped teats was much simpler than the model investigating potential causes of UCD. Only two explanatory variables remained in the model: peracetic acid in the pre-milking teat preparation and use of an automated dipping and flushing system. Peracetic acid is a common disinfectant used in the dairy industry and has not been linked to any major hypersensitivities or dermatitis in animals or humans unless used at high concentrations for prolonged periods (42–46). This is potentially useful information in that farmers can be made aware of the risk of teats becoming chapped in such situations and thereby increasing the risk of developing a case of ITN. In fact, a recent study found that using a flushing system with water alone, without the addition of peracetic acid, was effective in reducing bacterial numbers on the teat skin and may be a way to decrease the risk of ITN (47). Other potential interventions a farmer could take to reduce the incidence of chapped teats would be to use a post milking teat dip with a high emollient and perform a dynamic milking machine test, especially in the proposed high risk group of first lactation heifers. Whilst chapped teats in themselves may appear relatively minor problems, the potential subsequent increased risk of ITN should not be overlooked.

Although research into ITN is in its infancy, this study demonstrated several possible areas of intervention that farmers and veterinary surgeons could investigate should a case of ITN occur on a farm. Further studies are required to understand the potential for causality of these associated farm-level risk factors further, especially at the individual animal level. Furthermore, determination of disease aetiology and studies into the prevention and treatment of ITN is greatly needed. Whilst this study is only focused on GB farms, it highlights a disease that should be monitored in the rest of the world's dairy cow populations, especially given its severity and potential economic impact.

## Conclusions

Ischaemic teat necrosis appears to be reported more frequently in recent years and may cause substantial losses on dairy farms. Over half of the farmers that responded to this study had experienced a first case of ITN between 1985 and 2018. At the animal level, first lactation cows up to 90 days in milk are reported to be at the greatest risk of developing ITN. Farmer-reported potential farm-level risk factors for having cases of ITN on a farm were having cases of udder cleft dermatitis and/or chapped teats. These udder and teat presentations were found to have specific associated farm-level risk factors, which could be mitigated to improve teat health on farms.

## Data Availability Statement

The original contributions presented in the study are included in the article/Supplementary Material, further inquiries can be directed to the corresponding author/s.

## Ethics Statement

Ethical approval was granted by University of Liverpool, School of Veterinary Science Ethical Committee (application number: VREC460). The patients/participants provided their written informed consent to participate in this study.

## Author Contributions

HC-D, JWA, RB, AM, SC, and NE had major inputs in the study and questionnaire design, along with manuscript preparation. HC-D, JWA, SC, and NE conducted the data interpretation and analysis. JSA provided economic analysis and preparation of the manuscript. All authors contributed to the article and approved the submitted version.

## Funding

Research was funded by BBSRC Doctoral Training Partnership Studentship (BB/M011186/1) and a CASE Award from AHDB Dairy.

## Conflict of Interest

AM was employed by Quality Milk Management Services Ltd. The remaining authors declare that the research was conducted in the absence of any commercial or financial relationships that could be construed as a potential conflict of interest.

## Publisher's Note

All claims expressed in this article are solely those of the authors and do not necessarily represent those of their affiliated organizations, or those of the publisher, the editors and the reviewers. Any product that may be evaluated in this article, or claim that may be made by its manufacturer, is not guaranteed or endorsed by the publisher.

## Abbreviations

AHDB: Agricultural and Horticultural Development Board
BHM: bovine herpes mamillitis
CI: confidence interval
DD: digital dermatitis
DIM: days in milk
GB: Great Britain
ITN: ischaemic teat necrosis
lci-uci: lower confidence interval to upper confidence interval
OR: odds ratio
P: probability value
ROC: receiver operating curve
UCD: udder cleft dermatitis.
