# Supplementary material for: An Observational Study Investigating Potential Risk Factors and Economic Impact for Bovine Ischaemic Teat Necrosis on Dairy Farms in Great Britain
Source: Front Vet Sci. 2022 Mar 22;9:748259. doi: 10.3389/fvets.2022.748259 (PMC8981390; doi:10.3389/fvets.2022.748259)
Supplement: Supplementary file 4 [file Table_4.DOCX]

**Supplementary table 4. Probability of having ischaemic teat necrosis (ITN) in relation to the presence of udder cleft dermatitis (UCD) or chapped teats on the farm**: Predicted percentage probabilities from the final multivariable model.

| **UCD on Farm** | **Chapped teats on Farm** | **Predicted Percentage probability of having ITN from the model** | **Observed percentage of farms with ITN** |
| --- | --- | --- | --- |
| No | No | 37.8% (29.8-46.5%) | 37.6% |
| Yes | No | 63.0% (55.2-91.7%) | 63.2 % |
| No | Yes | 78.6% (51.1-73.4%) | 80.0% |
| Yes | Yes | 91.2% (76.0-97.1%) | 88.9% |
