# Supplementary material for: An Observational Study Investigating Potential Risk Factors and Economic Impact for Bovine Ischaemic Teat Necrosis on Dairy Farms in Great Britain
Source: Front Vet Sci. 2022 Mar 22;9:748259. doi: 10.3389/fvets.2022.748259 (PMC8981390; doi:10.3389/fvets.2022.748259)
Supplement: Supplementary file 7 [file Table_7.DOCX]

**Supplementary Table 7. Predicted probability of farm developing cases of chapped teats**: multivariable model with chapped teats as the outcome variable.

| **Peracetic acid in pre dip** | **ADF system use** | **Predicted Percentage probability of having chapped teats on the farm from the model** | **Observed percentage probability of having chapped teats on the farm** |
| --- | --- | --- | --- |
| No | No | 4.23% (1.38-12.3%) | 5.17% |
| No | Yes | 15.1% (6.51-31.4%) | 13.33% |
| Yes | No | 28.3% (10.0-58.3%) | 22.22% |
| Yes | Yes | 61.4% (26.6-87.5%) | 75.00% |
