# Supplementary material for: An Observational Study Investigating Potential Risk Factors and Economic Impact for Bovine Ischaemic Teat Necrosis on Dairy Farms in Great Britain
Source: Front Vet Sci. 2022 Mar 22;9:748259. doi: 10.3389/fvets.2022.748259 (PMC8981390; doi:10.3389/fvets.2022.748259)
Supplement: Supplementary file 8 [file Table_8.DOCX]

**Supplementary Table 8. The number and proportion of missing data for farmers reporting cases of udder cleft dermatitis (UCD) for the variables**: ischaemic teat necrosis (ITN), lactating cows bedded on sawdust, teat end eversion, and the time the calves are kept with the dams. Presented are the number of missing values due to the farmer not responding to the question and also due to the farmer responding with ‘don’t know’. The chi squared test was used to explore the associations between proportions of missing values and the outcome.

| **Variable** | **Missing and farmer did not report cases of UCD (%) n=140** | **Missing and farmer reported cases of UCD (%) n=77** | **p-value** |
| --- | --- | --- | --- |
| ITN | 0 (0.0%) | 0 (0.0%) |  |
| Didn’t know if had ITN | 0 (0.0%) | 0 (0.0%) |  |
| Lactating cows bedded on sawdust | 0 (0.0%) | 0 (0.0%) |  |
| Didn’t know if lactating cows were bedded on sawdust | 9 (6.4%) | 1 (1.3%) | 0.08 |
| Teat end eversion | 0 (0.0%) | 0 (0.0%) |  |
| Didn’t know if had teat end eversion | 34 (24.3%) | 19 (24.7%) | 0.95 |
| Time the calves were kept with the dams | 0 | 0 |  |
| Didn’t know how long the calves were kept with their dams | 3 (2.1%) | 2 (2.6%) | 0.83 |
