# Supplementary material for: An Observational Study Investigating Potential Risk Factors and Economic Impact for Bovine Ischaemic Teat Necrosis on Dairy Farms in Great Britain
Source: Front Vet Sci. 2022 Mar 22;9:748259. doi: 10.3389/fvets.2022.748259 (PMC8981390; doi:10.3389/fvets.2022.748259)
Supplement: Supplementary file 9 [file Table_9.DOCX]

**Supplementary Table 9. The number and proportion of missing data for farmers reporting cases of chapped teats for the variables**: peracetic acid used in the pre-milking teat preparation (pre dip), and the use of an automated dipping and flushing system (ADF). Presented are the number of missing values due to the farmer not responding to the question and also due to the farmer responding with ‘don’t know’. The Chi squared test or Fisher’s exact test, were appropriate, were used to explore the associations between the proportion of missing values and the outcome.

| **Variable** | **Missing and farmer did not report cases of chapped teats n=193** | **Missing and farmer reported cases of chapped teats n=24** | **p-value** |
| --- | --- | --- | --- |
| Peracetic acid in pre dip | 0 (0.0%) | 0 (0.0%) |  |
| Didn’t know if had peracetic acid in pre dip | 100 (51.8%) | 12 (50.0%) | 0.87 (Chi squared test) |
| Use an ADF system | 0 (0.0%) | 0 (0.0%) |  |
| Didn’t know if use an ADF system | 14 (7.2%) | 0 (0.0%) | 0.37 (Fisher’s exact test) |
