# Supplementary material for: An Observational Study Investigating Potential Risk Factors and Economic Impact for Bovine Ischaemic Teat Necrosis on Dairy Farms in Great Britain
Source: Front Vet Sci. 2022 Mar 22;9:748259. doi: 10.3389/fvets.2022.748259 (PMC8981390; doi:10.3389/fvets.2022.748259)
Supplement: Supplementary file 11 [file Data_Sheet_1.PDF]

The Disease- Bovine Ischaemic Teat Necrosis

We are first going to ask you some specific questions with regard to the disease. ***We are really interested in your answers even if you have not seen the disease.***

1. **Have you had any cases of cattle constantly LICKING their teats**? (Please circle)

Yes there is a current case | Yes I have seen it in the last month
Yes I have seen in the last year | Yes but it was more than one year ago
No, I have never seen this | Don't know

2. **Have you ever seen sores such as Fig. 1-4** (see Picture Guide provided) **in your dairy cattle?** (Please circle. If no or don't know, proceed to question 3 on page 4).

Yes No Don't know

We call these sores Bovine Ischaemic Teat Necrosis and from now on we will refer to them as ITN.

2a. Do you call these sores by any other name? (if so, please specify below)

………..

2b. Approximately, when did you FIRST SEE a case of Bovine ITN on your farm?

……………………………………………….................. month/year (please write approximate date)

2c. Roughly, how MANY TIMES in the last 5 years have you seen ITN lesions?

……….

2d. When the disease was at its worst roughly how MANY cases did you have at any one time?

……

2e. What LACTATION were the animals when you noticed the disease? (Please circle all that apply).

Pre-calving Heifers 1st Lactation 2nd Lactation

3rd Lactation 4th Lactation 5th Lactation 6th Lactation

Other, please specify……………………………………………… Don't know

2f. How far into lactation were the animals when you first noticed the disease? (Please circle)

1-30 days 30-90 days 90-200 days 200-300 days >300 days Dry Don't know

2g. If you have seen ITN, have you tried ANY treatments? (Please circle).

Yes (please specify all treatments attempted in **table** below and if you think they worked)

No (please move to question 2h)

	**Intramammary tube**	**Intramuscular injection**	**Subcutaneous injection**	**Administration on to skin** eg. Creams, sprays, washing etc.	**Other** eg. bull rings in nose, rubber rings around the neck etc
**Product used**					
**Did this work?** (yes/no/don't know)					
**What method worked the best?** (please tick/state)					

2h. What happened to the cows affected by ITN? (Tick for each case, this is a tally box, you can tick the box multiple times if relevant).

**Recovered**	**Retained the teat**			**Lost the teat**			**Culled**	**Other, please specify…………………**
	**Milked on 3**	**Did not get mastitis**	**Got mastitis**	**Milked on 3**	**Did not get mastitis**	**Got mastitis**		

2i. In which months have you EVER seen cases of ITN? (Please circle all that apply)

Jan Feb Mar April May June July Aug Sept Oct Nov Dec Don't know

2j. What time of year do you see the MOST cases of ITN? (Please circle all that apply)

Jan Feb Mar April May June July Aug Sept Oct Nov Dec Don't know

If you have any more specific information relating to cases of ITN and would be willing to share it, please if you might provide this information within the table on page 13.

The Udder

We are now going to ask some more general questions about the udders of your cows.

3. **In the last year HOW MANY cases of clinical mastitis** (e.g. hard udder, clots in milk) **have you had in the milking herd?** (a case is defined as one quarter affected once)

……….

4. **Do you know what agents have caused mastitis on your farm in the last year?** (i.e. has any milk testing done. Please write below if know the agent(s). You may write more than one).

……….

5. **How do you usually TREAT your mastitis cases?** (You many have more than one approach).

………

………

………

………

6. **What is your average Somatic Cell Count (SCC) for the last year?**

   ………

7. **In the last year approximately how many of the following lesions have you seen?** (Please use picture guide provided).

Lesion	Description	Number of cases	Current case? (Yes/No)
**Bovine Warts** (papillomavirus)	Wart or frond like lesions on the teat (**Fig. 5**)		
**Bovine Ulcerative Mammillitis** (Bovine herpesvirus 2)	Fluid filled blisters to large ulcerated regions along teats and on to udder skin (**Fig. 6-8**)		
**Udder Cleft Dermatitis**	Moist, pungent ulcerated areas on udder skin between the front quarters and between the udder and abdomen (**Fig. 9**)		
**Pseudocowpox/Milker's nodules** (parapox virus)	Red scabs that last 7-10 days and fall off to leave a **horseshoe/ring shape**. Causes milker's nodule in humans		
**Dermatitis/Udder acne**	Multiple raised scabs over the skin of the udder sometimes involving the teat (**Fig. 10**)		
**Chapped/dry skin** on teats	Dry skin on teats that is easily traumatised		

8. **Approximately <u>HOW MANY</u> cows in the herd have the following <u>after milking</u>?** (Please place a number in each column of the table).

**Discolouration of the teat**	**Swelling/ringing of the teat**	**Teat end hyperkeratosis**	**Teat end eversion**
Any discolouration of the teat (blue/pink/red) (**Fig. 11**)	Swelling of the teat of any kind (**Fig. 12**)	Raised/smooth roughened rings around the teat end (**Fig. 13**)	Streak canal everted after milking

9. **What is your usual <u>DRY COW protocol</u>?**

……

……

…….

10. **How <u>LONG</u> is your average cow's <u>dry period</u>?**

…………………………………………………………days

11. **What are your main <u>METHODS</u> for detecting clinical cases of <u>mastitis</u>?** (Please circle all that apply).

Clots in the milk | Other changes in milk | California Milk Testing (CMT)

Changes in the udder | Behaviour of cow | Hard quarter

Reduced milk yield | High/low temperature | Milk conductivity results

Being last/order come into the parlour | Other, please specify……………….

General questions about the cows on your farm

The following questions are related to the general health status of your dairy cattle.

12. **Have you ever seen Digital Dermatitis (also known as Digi, hairy heel warts, Mortellaro's disease.** See Fig. **14-15**) **in your cattle?** (Please circle).

    Yes, there are current cases — Yes I have seen it in the last month

    Yes I have seen in the last year — Yes but was more than one year ago

    No, I have never seen this (Please move to 13.) — Don't know (Please move to 13.)

    12 a. Approximately, when did you FIRST SEE a case of Digital Dermatitis?

    ……………………………………………………month/year (Please provide an approximate date)

    12 b. When the disease was at its worst roughly how MANY cases did you have at any one time?

    ……

    12 c. In which months have you EVER seen cases of Digital Dermatitis? (Please circle all that apply).

Jan Feb Mar April May June July Aug Sept Oct Nov Dec Don't know

12 d. What time of year do you see the MOST cases of Digital Dermatitis? (Please circle all that apply)

Jan Feb Mar April May June July Aug Sept Oct Nov Dec Don't know

13. **Have you had any animals persistently infected (PI) with Bovine Viral Diarrhoea Virus (BVDv) removed from the herd within the last year?** (Please circle).

    Yes No Don't know

14. **Do you VACCINATE your herd against BVDv and if so, WHAT PRODUCT do you use?**

………

15. **Have you had any cases of tuberculosis (TB) in the last 12 months?** (Please circle).

    Yes, confirmed case — Yes, reactor with no disease — No — Don't know

Milking Routine

You are doing great. Now we would like ask you a little about your milking routine and machine.

16. How many times a day do you milk your cows? (Please circle a letter).

A All cows are milked 2 times a day

B All cows are milked 3 times a day

C Cows are milked on a voluntary basis by an automated system (e.g. robot)

D Other, please specify……………………………………………………………………………………………….

17. Do you use a pre-milking teat dip/spray? (Please circle).

Yes No Don't know Other, please specify……………………………

17a. If you use a pre-milking teat dip/spray, WHAT do you use?

…………………………………………………………………………………………………

17b. How do you APPLY the product? (Please circle).

Spray Dip Foam cup Other, please specify…………………………….

18. Do you FOREMILK your cows (i.e. express milk before applying the cluster)? (Please circle).

Yes, always Most of the time Occasionally

Only if suspect mastitis No Don't know

19. How much TIME do you leave between preparing teats & attaching the cluster? (Please circle).

No time (clusters are attached immediately)

Less than 30 s 30 s to 1 min 1-2 min more than 2 mins

20. Do you wear disposable GLOVES when milking? (Please circle).

Yes, always Most of the time Occasionally No Don't know

21. Do you use paper towel to DRY the teats before milking? (Please circle a letter).

A Yes, new towel for each cow B Yes, but the same towel for multiple cows

C No, I let them dry naturally

D I use another method to dry the cow's teats. Please specify………………………………….

22. In what <u>ORDER</u> do you milk the <u>freshly calved</u> cows? (Please circle a letter).

A Before the rest of the milking herd

B After the milking herd but before cows with clinical mastitis

C After the rest of the milking herd

D They are milked with the milking herd in any order

E Other, please specify……………………………………………………………………………………..

23. In what <u>ORDER</u> do you milk the cows with <u>clinical mastitis</u>? (Please circle a letter).

A Before the rest of the milking herd

B After the rest of the milking herd

C They are milked with the milking herd, into a dump bucket with the same cluster

D They are milked with the milking herd, into a dump bucket using a separate cluster

E Other, please specify……………………………………………………………………………………..

24. What type of <u>PARLOUR</u> do you use? (Please circle).

Herringbone Rotary Swing Side by side Rapid exit Robotic

Other, please specify……………………………………….

25. Do you use an <u>Automatic Cluster Removal (ACR) system</u>? (Please circle).

Yes, it cuts out at…………………. No Don't know Other, please specify……………………..

26. Do you use an <u>Automated Dipping & Flushing (ADF)</u> system? (Please circle).

Yes No (Please move to 27.) Don't know (Please move to 27.)

26 a. If yes, what flushing fluid do you use? (Please circle).

Water Peracetic acid Other, please specify………………………..

27. Do you <u>DISINFECT</u> the clusters between cows? (Please circle).

Yes, between every cow Yes, but only if a cow has a case of mastitis

No Don't know Other, please specify…………………………………………….

27a. If yes, what disinfectant do you use? (Please state below).

……

28. What material are your <u>TEAT LINERS</u> made of? (Please circle).

Rubber Silicon Don't know Other, please specify…………………….

If possible, please can you provide the make and serial number…………………………..(this will allow us to determine the shape and size of the liners).

29. On average, after how <u>many</u> milkings/months do you <u>CHANGE</u> your teat liners?

……………………………………………………………………………milkings/months (please delete as appropriate)

30. How often is your milking machine <u>SERVICED</u>? (Please circle).

Every 1-5 months Every 6-9 months Every 10-12 months

Every 12-18 months When it needs it Never service the machine

31. Have you had a dynamic parlour test? (Please circle).

Yes, in the last 6 months Yes, in the last year Yes, but more than a year ago

No Don't know

31a. If Yes - Who performed your dynamic parlour test? (Please specify).

………

32. <u>WHO</u> milks the cows? (e.g. Yourself only, relief milkers, students, family members, hired staff)

………

33. Do cows <u>LEAK</u> milk before/after milking? (Please circle a letter).

A Yes there are cows that leak milk <u>before & after</u> milking (approximate number……………..)

B Yes there are cows that leak milk <u>before</u> they are milked (approximate number…………….)

C Yes there are cows that leak milk <u>after</u> they are milked (approximate number…………………)

D <u>None</u> of the cows leak milk before or after they are milked

E Don't know

Farm Environment

Nearly there! We just have a few more questions regarding your farm in general.

34. Do you keep any <u>other animals</u> on your farm? (i.e. Beef cattle, sheep, pigs, horses, dogs, and cats) (Please circle).

Yes, please specify……………………………………………………………………………………………

No, we keep only dairy cattle (please go to question 35.)

34 a. If you have sheep have you ever had a case of Contagious Ovine Digital Dermatitis (CODD) (Please see **Fig. 16** in the picture guide)?

Yes, currently   Yes, in the last 12 months   Yes but over 12 months ago

No   Don't know

35. Do you <u>BUY IN</u> any replacement dairy cattle? (Please circle a letter).

A Yes, directly from the <u>same farms</u> every time

B Yes, from <u>auction market</u>

C No, all replacements are <u>bred on farm</u> (please proceed to question 36)

D Other, please specify……………………………………………………………………………………….

35 a. Do you <u>ISOLATE</u> in a separate pen/field any dairy cattle brought on to the farm before introducing them into the herd? (Please circle).

Yes for……………….................. days/weeks/months (please delete as appropriate)

No   Don't know

36. Do you rear your heifers on site? (Please circle one or more letters).

A Yes, they are reared <u>on the same site</u> as the milking herd

B Heifers are reared on the <u>same farm</u> but on a <u>different site</u> to the milking herd

C Heifers are reared on a <u>separate farm</u>

D Don't know

E Other, please specify…………………………………………………

37. Are calving pens <u>shared</u> with sick or lame cows? (Please circle).

Yes   No   I don't know   Other, please specify…………………………………………

38. **Are freshly calved cows kept <u>separate</u> from the milking herd?** (Please circle).

Yes for …...........days/weeks　　No　　Don't know

39. **Please fill in the table below.** (Tick all that applies to each group of cows and fill in type of bedding if applicable).

	Lactating cows	Freshly calved cows	Dry cows	Heifers
Access to pasture for some of the year				
Housed at night				
Housed in winter only				
Housed year round				
Type of housing				
Cubicle houses				
Loose housing				
No housing				
Type of bedding material				
Please specify in each box				

40. **What <u>CATTLE BREEDS</u> are currently in your milking herd?**

………

41. **How <u>LONG</u> do calves stay with dams?**

…...............................hours/days (delete as appropriate)

42. **Do you use <u>artificial insemination (AI)</u>, <u>embryo transfer (ET)</u>, <u>bull</u> or a <u>mixture?</u>** (Please circle).

AI　　ET　　Bull　　Mixture, please specify…………………………………………………………………

42 a. What are your current priorities when selecting an AI bull?

………

43. **When do you calve?** (Please circle).

Spring only　　Spring and autumn　　Autumn only　　Year round

Other, please specify……………………………………………………………….

43a. If you calve <u>year round</u>, what is your average <u>NUMBER</u> of cows in milk and dry?

Cows in milk…………….　　Cows dry…………………

44. **How <u>MANY</u> cattle do you have and what is the <u>SIZE</u> of your milking herd?** (Please state number in each category).

Total cattle…………………………………. (including beef cattle, dairy bull calves etc.)

Total milking herd………………………..

45. **What is your cows' average <u>LENGTH of lactation</u>?** (Please state number).

…………………days

46. **What is your average <u>MILK YIELD</u> per cow <u>per lactation</u>?**

………

47. **And lastly what <u>county/counties</u> is your farm in?**

………

Please use the space below for anything you would like to tell us that you feel is important for us to consider, or if you feel there is anything we may have overlooked.

………
………
………
………

If you wish to be entered into a prize draw for the chance to win a £100 Amazon voucher and to receive the results of this national survey please fill in your name and contact details below:

Name:

Address:

Postcode:

Email:

Telephone:

Would you be happy for us to contact you in the future about your questionnaire answers? (Please circle your response). Yes No

<u>THANK YOU FOR YOUR TIME IN PARTICIPATING IN THIS QUESTIONNAIRE!</u>

<u>Please note that your response has potentially helped hundreds of other dairy farmers and thousands of cattle throughout the UK!</u>

If you are willing to share any specific information on cows that have had ITN please fill in the table below.

Date Calved	Date first saw the lesion	Lactation number	Details of the case
E.g 29/08/2017	14/09/2017	1	Sore on teat, constantly licking, lost teat. Tried udder cream, no response.
